# Secondary hemophagocytic lymphohistiocytosis associated with Rocky Mountain spotted fever in a toddler: A case report

**DOI:** 10.1002/jha2.405

**Published:** 2022-04-05

**Authors:** Smitha Hosahalli Vasanna, Peter Paul C. Lim, Tanya Saeeda Khan, Jignesh Dalal

**Affiliations:** ^1^ Department of Pediatric Hematology‐Oncology Rainbow Babies and Children's Hospital/ University Hospitals Cleveland Ohio USA; ^2^ Department of Pediatric Infectious Diseases Rainbow Babies and Children's Hospital/University Hospitals Cleveland Ohio USA; ^3^ Department of Pediatrics Rainbow Babies and Childreny's Hospital/University Hospitals Cleveland Ohio USA

**Keywords:** Hemophagocytic lymphohistiocytosis, *Rickettsia rickettsii*, Rocky Mountain spotted fever

## Abstract

A three‐year‐old boy presented with fever, maculopapular rash involving palms and soles, and hyponatremia two weeks following a tick bite. Empiric doxycycline that he was on was discontinued following negative initial rickettsial serology based on the non‐endemicity of Rocky Mountain spotted fever (RMSF) in Northeast Ohio. He demonstrated high inflammatory markers and met the criteria for hemophagocytic lymphohistiocytosis (HLH). With a working diagnosis of macrophage activation syndrome secondary to presumed systemic‐onset juvenile idiopathic arthritis (soJIA), he received HLH‐directed therapy. Rising antibody titers in convalescent sera established the diagnosis of RMSF. The patient recovered completely with HLH directed therapy and re‐institution of doxycycline. This is the first pediatric case report of *Rickettsia rickettsii* induced HLH demonstrating a favorable outcome despite modified therapy.

## 1 BACKGROUND

Rickettsial infections are known to cause secondary HLH. In North America, RMSF is the most prevalent and severe Rickettsial tick‐borne illness associated with a 20–30% mortality in untreated cases [1, 2]. Rickettsia‐related HLH caused by scrub typhus, Mediterranean spotted fever (MSF), and ehrlichiosis have been reported in the literature [3, 4, 5, 6]. Despite RMSF being the most common rickettsial infection, there have been no published reports of HLH secondary to it aside from two studies describing RMSF associated HLH on postmortem cases [7, 8]. Here, we report a case of a toddler with HLH due to RMSF from Northeast Ohio, USA who recovered well on appropriate therapy.

## 2 CASE PRESENTATION

A healthy, fully immunized 3‐year‐old boy presented with high‐grade persistent fever and rash for 5 days. The rash started on his legs but soon became generalized, spreading from head to toe along with the involvement of palms and soles. The rash was non‐pruritic, non‐vesicular, non‐tender without desquamation. The limp was evident due to swollen and painful ankle joints. A tick was noted at the nape of his neck while playing in the woods two weeks prior to presentation. It was not engorged and was totally removed, though his dad was unsure of the tick type. There was no history of travel, sick contact, or sickness of the family dog.

**FIGURE 1:**
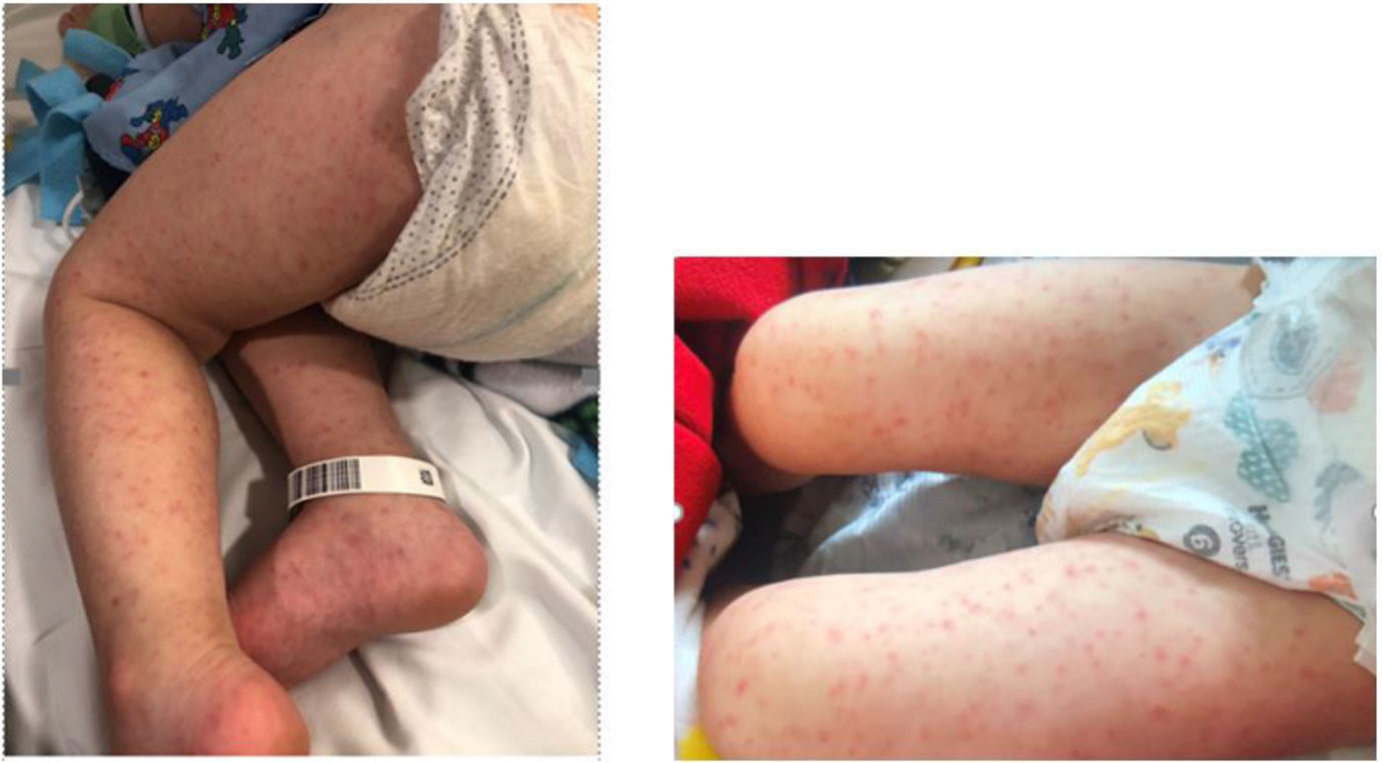
Maculopapular/purpuric rash

On exam, he was febrile to 40.2° C with tachycardia. Other vitals were normal. He was extremely irritable with mild periorbital puffiness, generalized non‐blanchable maculopapular/purpuric rash (as depicted in Figure 1) including palms and soles. Redness of lips was noted without any pharyngeal hyperemia/ strawberry tongue or scleral injection. No lymphadenopathy or hepatosplenomegaly was noted. His cardiac and pulmonary exams were normal. Neurologic exam was significant for irritability, without overt signs of meningismus. Bilateral ankle joints were swollen with tenderness to palpation. Laboratory results are elaborated in Table 1.

**TABLE 1:** Lab parameters and imaging

Test	Results	Reference Range
CBC	Hemoglobin 10 gm/dl (day 1 of admission), 8.8 gm/dl (day 3 of admission) WBC 9.5 × 10^9^/L Platelets 117 × 10^9^/L (day 1 of admission), 52 × 10^9^/L (day 3 of admission)	11.5–13.5 5.0–17.0 150–400
RFP	BUN 8 mmol/L Creatinine 0.27 mg/dl Sodium 122 mmol/L	6–23 0.2–0.5 136–145
LFT	Aspartate aminotransferase (AST) 128 U/L Alanine aminotransferase (ALT) 60 U/L Total bilirubin (TB) 0.5 mg/dl Direct bilirubin (DB) 0.1 mg/dl	16–40 3–28 0–0.7 0–0.3
Inflammatory markers	ESR 31 mm/h CRP 3 mg/dl (day 1 of admission) to 11 mg/dl (day 2 of admission) Serum ferritin 1355 D‐dimer 15698	20–300 <1 20–300 <500
CSF studies Rapid Biofire	Normal Glucose, Protein, Negative Gm stain and Cultures Negative	
EBV and CMV PCR, HSV 1 and 2, HHV‐6 PCR, Lyme Antibody Titers	Negative	
Rocky Mountain Spotted Fever (RMSF), Ehrlichia, Anaplasma titers	< 1: 64	<1:64
Histoplasma Ag	Not detected	
Anti‐nuclear antibodies reflex to ENA	Negative	
Ultrasound (US) joints Abdominal US	Bilateral ankle joint effusions Hepatomegaly and moderate amount of free peritoneal fluid and small right‐sided pleural effusion	
Echocardiogram	No abnormalities	

Rickettsial infection especially with the history of tick bite and Meningococcal meningitis were the top differentials, and hence he was initiated on empiric doxycycline and ceftriaxone.

On the second day of hospitalization, he developed hypotension with rising inflammatory markers and worsening bicytopenia and was transferred to the intensive care unit. Additional differentials considered at this point included atypical Kawasaki, Macrophage activation Syndrome (MAS) secondary to soJIA, multi‐system inflammatory syndrome in children (MIS‐C), and HLH. Our patient met 6/9 HLH criteria (fever, bicytopenia, hyperferritenemia, hypertriglyceridemia 301 mg/dl [ref range (RR) 0‐149], high soluble IL‐2: 4004 pg/ml (RR: 75.3‐858.2), and CXCL9:842 pg/ml (RR: < = 121). Due to worsening clinical status, he received IVIG 1 g/kg, methylprednisolone 30 mg/kg, and anakinra for presumed HLH/MAS. Due to negative RMSF serology, in addition to the non‐endemicity of RMSF in Northeast Ohio and, negative travel history, doxycycline was discontinued on the third day of antimicrobial therapy. Systemic steroids and anakinra were continued with probable diagnosis of MAS secondary to soJIA.

Given his young age and high Hscore [9], 166 points with a 40–54% probability of HLH, a genetic panel was sent to rule out primary HLH which showed LYST C.9925+9A > C (Intronic) heterozygous variance of unknown significance (VUS). Chediak Higashi Syndrome was considered unlikely, especially in the absence of other suggestive clinical features and heterozygous VUS LYST mutation. His clinical condition eventually improved, and he was discharged home on daily anakinra 1 mg/kg and tapering course of steroids.

At two weeks follow up, he had persistent rash and intermittent joint pain. Because of the history of tick bite, suspicion for RMSF was reconsidered based on the clinical course. Repeat antibody titers were sent in the convalescent sera, which showed 1:1024 titers for both RMSF IgM and IgG strongly compatible with recent RMSF. Since our patient had not completed a full course of doxycycline, additional 7 days of treatment was given. His symptoms were fully resolved at 4 weeks follow‐up with normal inflammatory markers and blood counts. Anakinra was also eventually stopped after few weeks. A final diagnosis of RMSF induced secondary HLH was made.

## 3 DISCUSSION

Although our patient had fever, classical rash, hyponatremia, and tick bite suggestive of RMSF, negative serology and disease epidemiology played heavily in the therapeutic decision making. Epidemiologically, most of the cases in Ohio come from the southern portions of the state and there were no reported cases of RMSF in Northeastern Ohio since 2011 [10, 11].

Serologic tests have low sensitivity during the first 14 days of illness. If the initial serologic test is negative, it should be repeated between 14 and 21 days when the sensitivity is around 94%. The only reliable test that may confirm the diagnosis within 14 days of illness is a skin biopsy of the lesion which can be sent for immunohistochemical staining or direct PCR testing, although this test is not routinely recommended due to its invasive nature [12].

RMSF has a mortality rate of approximately 80% if treatment is delayed after five days of illness. Thus, empiric therapy with doxycycline should be initiated if the clinical suspicion is high and should be completed even with negative serology [13]. The gold standard test for the diagnosis of Rickettsial diseases according to CDC and WHO is the four‐fold rise of immunoglobulin titers in the convalescent serum [1].

HLH is a life‐threatening condition characterized by severe cytokine storm secondary to immune dysregulation. The exact pathophysiology of Rickettsial HLH is not fully understood. *R. rickettsii* primarily infects endothelial cells causing vasculitis and in severe cases thrombosis and hemorrhage. An interesting postulate is that this endothelial injury is neutrophil‐mediated leading to the release of Interleukin (IL)‐8 and other inflammatory mediators in high proportions in the case of RMSF. A fatal case of RMSF in a 36‐year‐old lady who had very high quantities of TNF‐α, IL‐6, IL‐8, and intercellular adhesion molecule‐1 (ICAM‐1) concentration was reported by Sessler et al. [14]

There are no clear guidelines on the treatment of infection‐associated HLH. Among several published case reports of secondary HLH due to Rickettsial infection, antimicrobials are considered the cornerstone of therapy. Kaneko et al performed the PubMed search for HLH cases with rickettsiosis and identified 27 cases from 15 case reports: 21 cases of *Orientia tsutsugamushi*, four of *Rickettsia conorii*, and one of *Coxiella burnetti*. Twelve of these cases were treated with rickettsia specific antibiotics, two cases received antibiotics with immunoglobulin (IVIG), eight were given antibiotics with systemic steroids with/without IVIG, two were given antibiotics with systemic steroids and chemotherapy (etoposide, and/or cyclosporine), and treatment was unknown in three cases. Twenty‐three cases survived and four cases died [15]. HLH secondary to infection has a favorable outcome provided the infection is appropriately treated in a timely fashion. The expert consensus strongly recommends only antibiotic therapy in infection‐associated HLH, especially in mild cases, though moderate to severe cases might also warrant HLH directed therapy in addition to antibiotics.

Though our patient was only partially treated with rickettsia‐directed antibiotic initially, immunomodulatory therapy including steroids, IVIG and Anakinra likely dampened the cytokine storm leading to partial improvement. His arthritis and rash fully resolved only after the completion of delayed doxycycline treatment.

Our case highlights the importance of continuing rickettsia‐directed antibiotic therapy when there is a strong clinical suspicion even when initial serology is negative. If in doubt, a skin biopsy may be performed rather than discontinuation of antibiotics due to high reported mortality with RMSF infections with HLH.

## CONFLICT OF INTEREST

The authors declare no conflict of interest.

## ETHICS STATEMENT

IRB approval obtained and consent obtained.

## FUNDING

None.
